# Overexpression of Nuclear Apoptosis-Inducing Factor 1 Altered the Proteomic Profile of Human Gastric Cancer Cell MKN45 and Induced Cell Cycle Arrest at G1/S Phase

**DOI:** 10.1371/journal.pone.0100216

**Published:** 2014-06-13

**Authors:** Mei Yang, Jialing Zhong, Mei Zhao, Jia Wang, Yuyu Gu, Xinghua Yuan, Jianli Sang, Changzhi Huang

**Affiliations:** 1 Department of Etiology and Carcinogenesis and State Key Laboratory of Molecular Oncology, Cancer Institute and Hospital, Chinese Academy of Medical Sciences and Peking Union Medical College, Beijing, P.R. China; 2 Department of Abdomen Surgery, Cancer Institute and Hospital, Chinese Academy of Medical Sciences and Peking Union Medical College, Beijing, P.R. China; 3 Institute of Cell Biology, College of Life Sciences, Beijing Normal University, Beijing, P.R. China; Ospedale Pediatrico Bambino Gesu', Italy

## Abstract

Nuclear apoptosis-inducing factor 1 (NAIF1) was previously reported to induce apoptosis. Moreover, the expression of NAIF1 was significantly down-regulated in human gastric cancer tissues compared to adjacent normal tissues. However, the mechanism by which the NAIF1 gene induces apoptosis is not fully understood. Our results show that NAIF1 was minimally expressed in all the tested gastric cancer cell lines. Our data also demonstrates that NAIF1 is localized in the nuclei of cells as detected by monitoring the green fluorescence of NAIF1-GFP fusion protein using fluorescent confocal microscopy. Next, a comparative proteomic approach was used to identify the differential expression of proteins between gastric cancer cell lines MKN45/NAIF1 (−) and MKN45/NAIF1 (+). We found five proteins (proteasome 26S subunit 2, proteasome 26S subunit 13, NADH dehydrogenase Fe-S protein 1, chaperonin containing TCP1 subunit 3 and thioredoxin reductase 1) that were up-regulated and three proteins (ribonuclease inhibitor 1, 14-3-3 protein epsilon isoform and apolipoprotein A-I binding protein) that were down-regulated in the MKN45 cells overexpressing NAIF1. We also discovered that NAIF1 could induce cell cycle arrest at G1/S phase by altering the expression of cell cycle proteins cyclinD1, cdc2 and p21. The differentially expressed proteins identified here are related to various cellular programs involving cell cycle, apoptosis, and signal transduction regulation and suggest that NAIF1 may be a tumor suppressor in gastric cancer. Our research provides evidence that elucidates the role of how NAIF1 functions in gastric cancer.

## Introduction

Gastric cancer is one of the most common malignancies in the world causing approximately 8% and 10% of annual cancer cases and deaths, respectively. According to the world-wide epidemic report by the World Health Organization, nearly one million gastric cancer cases and 738,000 deaths are estimated to have occurred in 2008 [1], [2]. Many efforts have been taken in clinical; however, the mortality of gastric cancer patients is still as high as 70% [2]. One reason for this high mortality is that gastric cancer patients are often not diagnosed until the advanced stage, which is too late to provide effective treatment. Hence, there is an obvious need to find new bio-markers and effective strategies for early diagnosis and treatment of gastric cancer.

Proteomics has been used in many research areas, including cancer research. Common samples in proteomic analysis for cancer research include tissue and blood from cancer patients, as well as cancer cell lines with different backgrounds or different treatments [3]–[6]. These proteomic analyses were used to investigate the origination and development of cancer or to look for diagnostic biomarkers. The results we obtained through proteomic methods are not only due to direct regulation of transcriptional level, but also reflect post-translational modifications of proteins [3], [7]. Therefore, we can analyze both expression and regulation of protein with proteomic analyses. Despite lots of emerging techniques, 2-dimensional electrophoresis coupled with mass spectrometry has remained the most utilized method for proteomic analysis.

The human gene encoding nuclear apoptosis-inducing factor 1 (NAIF1) is located on chromosome 9q34.11. NAIF1 encodes a protein with a *Myb*-like domain at its N-terminal region [8], [9]. Previous studies have shown that overexpression of NAIF1 in human cancer cell lines HeLa and MKN45 induces apoptosis [8], [10]. Moreover, Luo *et al.* found that NAIF1 is significantly expressed in normal gastric tissue, while its expression is down-regulated or lost in gastric cancer tissues (*P<*0.001). In addition, NAIF1 expression was higher in well-differentiated (*P* = 0.004) than in moderately- or poorly-differentiated gastric cancer [10]. However, the role of NAIF1 in regulating the cellular protein expression profile is unknown.

In the present study, we employed proteomic technology based on 2-dimensional electrophoresis combined with MALDI-TOF mass spectrometry to identify proteins associated with the biological functions of NAIF1. The protein expression profiles of the gastric cancer cell line, MKN45 with or without NAIF1 overexpression were analyzed and compared using 24 cm pH 3–10 NL range immobilized pH gradient (IPG) strips. To validate this data, we measured RNA expression levels of all the differently expressed proteins and three proteins were further verified by western blotting. Our results demonstrate that NAIF1 induces cell cycle arrest at G1/S phase by regulating the expression levels of cyclinD1, cdc2 and p21 protein. The results of our study may lead to a better understanding of how NAIF1 works in inducing apoptosis and also may shed light on the diagnosis and therapy of gastric cancers in the future.

## Materials and Methods

### Cell culture and transfection

Human gastric cancer cell lines MKN45, BGC823, AGS and SGC7901 were obtained from the Tumor Cell Bank of Chinese Academic of Medical Sciences and cultured in liquid Dulbecco's minimum Essential medium (DMEM) supplemented with 10% heat-inactivated fetal bovine serum (FBS), 100 IU/ml penicillin, 100 µg/ml streptomycin at 37°C in a humidified 5% CO_2_ atmosphere. DNA transfection was performed using the X-tremeGENE HP DNA transfection reagent (Roche, Switzerland) according to the manufacturer's instructions.

### Expression plasmids

The expression plasmids pEGFP-N1-NAIF1, which expresses a NAIF1-GFP fusion protein, and pEGFP-N1, which expresses green fluorescent protein (GFP), was kindly provided by Dr. Qing Luo [10].

### Cell cycle distribution analysis

Exponentially growing cells were transfected with the pEGFP-N1 vector or the pEGFP-N1-NAIF1 plasmid. Cells were harvested at 24 h or 48 h post transfection, and 1×10^6^ cells were washed twice with phosphate-buffered saline (PBS) and fixed with 4% paraformaldehyde at 4°C for 1 h. Following centrifugation, the cell pellets were re-suspended in staining solution (0.05 mg/ml propidium iodide (PI), 0.2 mg/ml RNase, and 0.1% Triton X-100) at 4°C for 15 min in the dark. Flow cytometry analysis for GFP and PI was performed using a BD LSR II cell analyzer (BD, San Jose, CA, USA). PI fluorescence was measured among the GFP positive cell population and the distributions of the cell cycles were analyzed using ModFit LT3.2 software. Three separate samples were prepared and all assays were performed in triplicate.

### Quantification of apoptosis

Quantification of apoptosis was performed using the PE Annexin V Apoptosis Detection Kit I (BD). Briefly, BGC823 or MKN45 cells were transfected with the pEGFP-N1 vector or the pEGFP-N1-NAIF1 plasmid. Following 24 h or 48 h post transfection, the cells were harvested, washed with cold PBS twice and then re-suspended with binding buffer (10 mM Hepes/NaOH (pH 7.4), 140 mM NaCl, 2.5 mM CaCl_2_). The re-suspended cells were incubated with Phycoerythrin (PE) conjugated Annexin V (AV) and 7-Amino-Actinomycin (7-AAD), and then measured by flow cytometry using the BD LSR II cell analyzer. Four distinct cell populations could be detected in a dot-plot: viable cells (AV^−^/7-AAD^−^), early-phase apoptotic cells (AV^+^/7-AAD^−^), late-phase apoptotic cells (AV^+^/7-AAD^+^), and necrotic cells (AV^−^/7-AAD^+^). A minimum of 10,000 GFP positive cells were counted per sample and the data was reported as the percentage of apoptotic cells (AV^+^/7-AAD^−^ and AV^+^/7-AAD^+^) among total cells. Three independent sample preparations were made and all assays were repeated in triplicate.

### Confocal scanning

Forty-eight hours post transfection, cells were washed three times with PBS and fixed with 4% paraformaldehyde for 15 min. at room temperature. To enhance the permeability of the cell membrane, cells were incubated with 0.5% Triton X-100, followed by incubation with 4′, 6-diamidino-2-phenylindole (DAPI) to stain the nuclei. Cellular distribution of NAIF1 was analyzed by monitoring the green fluorescence of the NAIF1-GFP fusion protein using a Leica Microsystems Heidelberg GmbH microscope.

### Protein preparation for 2-DE

For each sample, 5×10^6^ cells were harvested and lysed in 1 ml lysis buffer (7 M urea, 2 M thiourea, 2% CHAPS, 20 mM DL-Dithiothreitol (DTT), 1 mM phenylmethylsulfonylfluoride (PMSF), 30 mM sodium fluoride, and 0.25 mg/ml RNaseA) for 1 h at room temperature, and then centrifuged at 4°C for 30 min. at 12,000 rpm. The supernatant was transferred to a new tube and concentrated with 0.1 M NH_4_COOH. Lysis buffer was added to a final volume of 800 µl, and the protein concentration was determined using a Bradford assay (Tiangen, Beijing, China).

### Two-dimensional electrophoresis

Two-dimensional electrophoresis was performed as previously described [5]. In brief, 1 mg of proteins was diluted to 450 µl with rehydration buffer (7 M urea, 2 M thiourea, 2% CHAPS, and 20 mM DTT). IPG strips (IPG, 24 cm, pH 3–10, NL, GE Limit.) were rehydrated with the rehydrated samples for 18 hours at room temperature. The proteins were subjected to isoelectric focusing with a Ettan IPGphorIII (GE, CT, USA) successively for 1 h at 100 v, 1 h at 250 v, 1 h at 500 v, 1 h at 1000 v, 1 h at 3000 v, 1 h at 5000 v, 2 h at 8000 v, and 6 h at 8000 v to get a total of 80 kvh. Following the isoelectric focusing, IPG strips were equilibrated with equilibrating buffer I (6 M urea, 150 mM Tris-HCl (pH 8.8), 30% glycerol, 2% SDS, 1% DTT) for 15 min. followed by a second incubation with another equilibrating buffer II (6 M urea, 150 mM Tris-HCl (pH 8.8), 30% glycerol, 2% SDS, 2.5% iodoacetamide) for 15 min. The second-dimension of SDS-PAGE was carried out with 12.5% SDS-PAGE gels using the Ettan DALT SIX vertical systems (GE).

### Gel staining and image analysis

The SDS-PAGE gels were fixed in fixing-buffer (10% CH_3_COOH, 40% C_2_H_5_OH, and 50% ddH_2_O) for 30 min. The gels were then washed in ddH_2_O for 10 min. 3 times and stained with Coomassie blue G-250.

The gels were scanned with an image scanner using MagicScan software to evaluate the spot patterns. Imagemaster platinum software (version 5.0) was employed to analyze the gel images. The spots which were observed on both the control gel and NAIF1 gel were analyzed and the intensity of each spot was quantified by calculating the spot volume after normalizing the gel image. Quantitative analysis of gel images (three replicates/sample) were taken and spots with statistically significantly differences (t-test, *P<0.05) were excised and digested for identification.

### Protein identifications

Differentially expressed protein spots were extracted and de-stained in 50 mM NH_4_HCO_3_/acetonitrile (50/50) and dried by vacuum centrifugation. The gel pieces were then digested with 10 ng/ml trypsin (Promega, WI, USA) in 50 mM NH_4_HCO_3_ buffer at 37°C overnight. The trypsin liquid was transferred to a clean tube, diluted with 100 µl 60% acetonitrile/0.1% trifluoroacetic acid, and treated with ultrasound for 15 min, three times. All the acquired liquid was collected and vacuum dried and stored at −80°C. The eluted peptides were analyzed using a Bruker ultraflex MALDI-TOF/TOF equipped with the SCOUT source by BGI-Beijing. The results of mass spectrum were contrasted against mammalian sequences from the NCBInr (non-redundant) database for peptide mass fingerprinting identification.

### RNA Isolation, RT and Real-time Q PCR

Total RNA was extracted using TRIzol reagent (Invitrogen) according to the manufacturer's protocol. cDNA was synthesized by reverse transcription using 1 µg total RNA with oligo (dT)_18_ primers and M-MuLV reverse transcriptase (TAKARA).

For reverse transcriptional PCR, primers for NAIF1 and β-actin were as follows: NAIF1 sense, 5′- AGCCACGGTCACCCTGACACA-3′, and anti-sense, 5′- CGTGTCTGCATGCTGGGCCAT-3′; β-actin sense, 5′- GGGCACGAAGGCTCATCATT-3′, and anti-sense, 5′- AGCGAGCATCCCCCAAAGTT. All the primers were designed using NCBI blast and purchased from Sangon, Beijing.

Quantitative PCR was performed with the SYBR Green qPCR Kit (TAKARA, Japan) with a 20 µl reaction system set up according to the manufacturer's instructions. Target gene expression was analyzed using appropriate real-time qPCR primers (Table 1). β-actin was used for normalization. The real-time qPCR was performed on an ABI7300 cycler (ABI, MA, USA) with the following conditions: denaturation for 30 s at 95°C, followed by 40 cycles of denaturation for 5 s at 95°C, and extension for 31 s at 60°C. Melting curve analysis was performed at the end to validate the specificity of the expected PCR product. Relative expression was calculated using the two standard curve methods. Three independent samples were prepared for each condition and each experiment was performed in triplicate.

**Table 1:** Primer information of real-time Q PCR.

Official symbol	Forward primer(5′–3′)	Reverse primer(5′–3′)	Annealing temperature/°C
NDUFS1	TCGGATGACTAGTGGTGTTA	TTATAGCCAAGGTCCAAAGC	60
TXNRD1	TCCTATGTCGCTTTGGAGTGC	GGACCTAACCATAACAGTGACGC	60
TCP1-γ	TGGGGCCCGGATAGTCAGCC	TGGTGCAGGCCTTGGGGTCT	60.04
PSMC2	AGGGATGAGAGTGGGCGTGG	ACCTGCATCATGGTAACTGTTGGG	57.81/57.14
RNH1	CGGGACCTGTGCGGCATTGT	GAGCCTGGAGCTGGGGTGGA	59.97/59.89
PSMD13	ACACGAAGAAGTTGTGGCATCAGC	TCTCCTTGGGCAAAGCACGGA	58.21/58.35
14-3-3ε	AGCCGCTGCCGCTATGGATGA	CTGCGGGTTCAGTTCCAGAGCAC	60.71/60.00
APOAIBP	AAATGGACATCCCTTTCCTTGG	TTCCCGAACATCGCCCTTG	60.2/62.3
ACTIN	CTGGAACGGTGAAGGTGACA	AAGGGACTTCCTGTAACAATGCA	60
NAIF1	ACGGTCACCCTGACACAGATCCC	GCGGCTTGACCGACGTGTCT	59.93/59.71

### Western Blotting

Western blotting was performed as previously described. Briefly, 2×10^6^ cells were harvested with medium flow, pelleted by centrifugation for 5 min at 500 *g*, and washed 3 times with PBS. The cells were then lysed with 100 µl lysis buffer (50 mM Tris-HCl, pH 7.4, 150 mM NaCl, 1.5% NP-40, 0.1% SDS, and 50 µg/ml PMSF, with freshly added proteinase inhibitor cocktail) on ice for 30 min. Lysates were centrifuged at 13,000 rpm for 15 min. and the protein concentration was measured using Bradford methods. Fifty micrograms proteins was separated on 12% SDS-PAGE and transferred to polyvinylidene difluoride membranes. The membranes were blocked with 5% BSA in TBST for one hour at room temperature followed by incubation with primary antibody solutions according to the manufacturer's instructions (β-actin, 1∶5000, Sigma; NAIF1, 1∶1000, Santa Cruz; 14-3-3 ε, 1∶500, Santa Cruz; TCP1-γ, 1∶1000, Abcam; TXNRD1, 1∶1000, Abcam; CyclinD1, 1∶1000, CST; cdc2, 1∶500, CST; and p21, 1∶500, CST). The membranes were washed 3 times with TBST, followed by incubation with appropriate secondary antibodies for two hours at room temperature. The signals were detected using the ECL Chemiluminescence system. β-actin was employed as the control.

### Statistical Analysis

All statistical analyses were performed using SPSS 13.0 software (SPSS Inc., Chicago, IL, USA). Data were expressed as means ±SD. The student's t-test was used for statistical comparison. An associated value of *P<*0.05 was considered significant.

## Results

### Transfected NAIF1 localized in the nucleus

**Figure 1:**
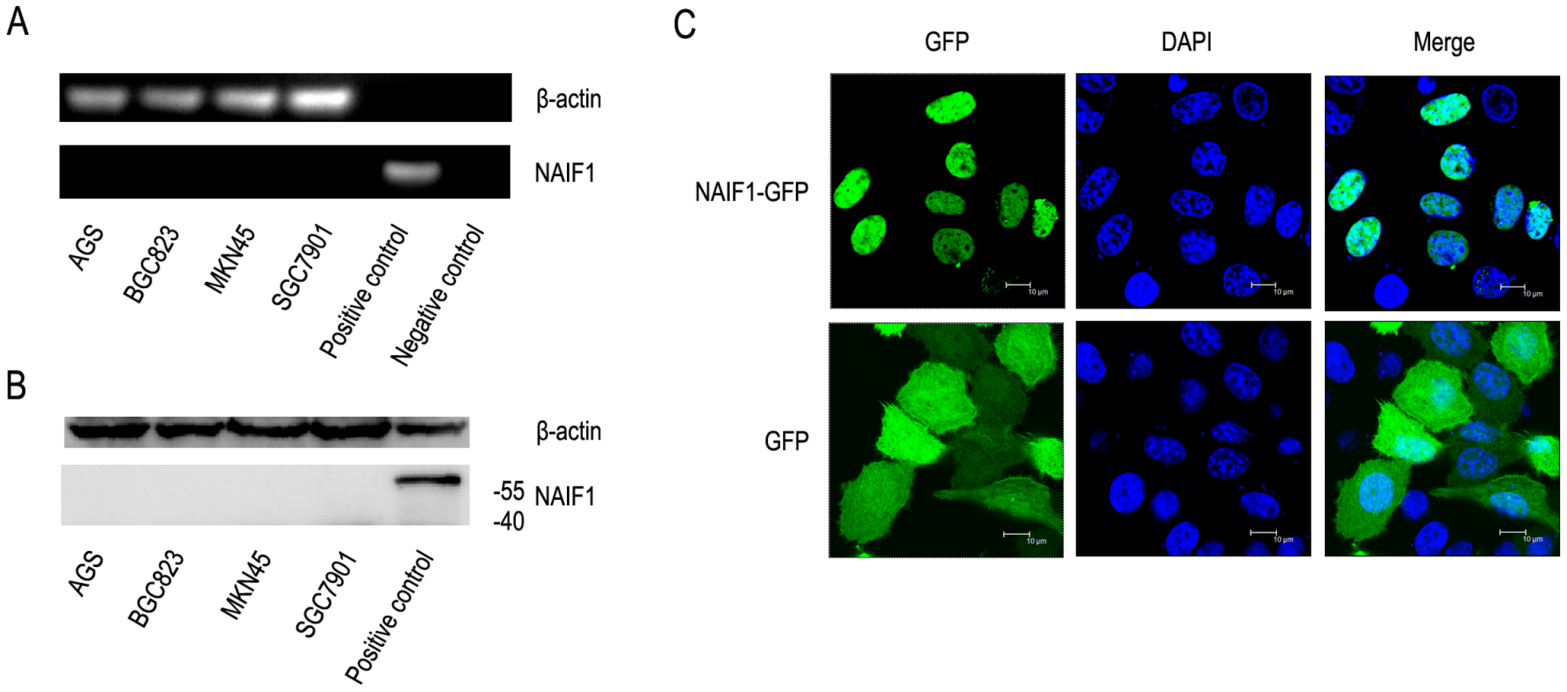
Expression and localization of NAIF1. (A) Reverse transcriptional PCR shows NAIF1 is not expressed at the RNA level in the tested cells. Plasmid containing the NAIF1 gene was used as a template for the positive control and ddH_2_O was used as the template for negative control. (B) Western blotting showed that NAIF1 is not expressed at the protein level in the four tested gastric cancer cell lines. MKN45 cells overexpressing NAIF1-GFP fusion protein was used as the positive control. The transfected NAIF1 band was detected between the protein molecular weight markers 55 and 75 kD; while no bands were detected between 40 and 55 kD, which is the predicted position of the NAIF1 protein. (C) Subcellular distribution of NAIF1. MKN45 cells were transfected with either the NAIF1-GFP fusion construct or the GFP vector. The upper three pictures represent the NAIF1-GFP fusion protein is distributed only in the nuclei, while the pictures below represent GFP is distributed throughout the entire cell.

Both reverse transcriptional PCR and western blotting experiments demonstrated that NAIF1 was minimally expressed in gastric cancer cell lines, MKN45, BGC823, AGS, and SGC7901; whereas NAIF1 was easily detected when it was transfected into the cells (Figure. 1A and B). We transfected a NAIF1-GFP fusion construction into MKN45 and BGC823 cells and a GFP vector was used as the control. Confocal microscopy demonstrated that when cells were transfected with the pEGFP-N1-NAIF1 construct, green fluorescence was detected only in the nuclei; on the contrary, the green flurescent signal was detected throughout the entire cell when transfected with the GFP vector (Figure. 1C). The same results were also observed in other cell lines such as BGC823 (Figure S1). These findings confirmed that transfected NAIF1 protein is localized in the nucleus.

### NAIF1 induced cell cycle arrest at G1/S phase

**Figure 2:**
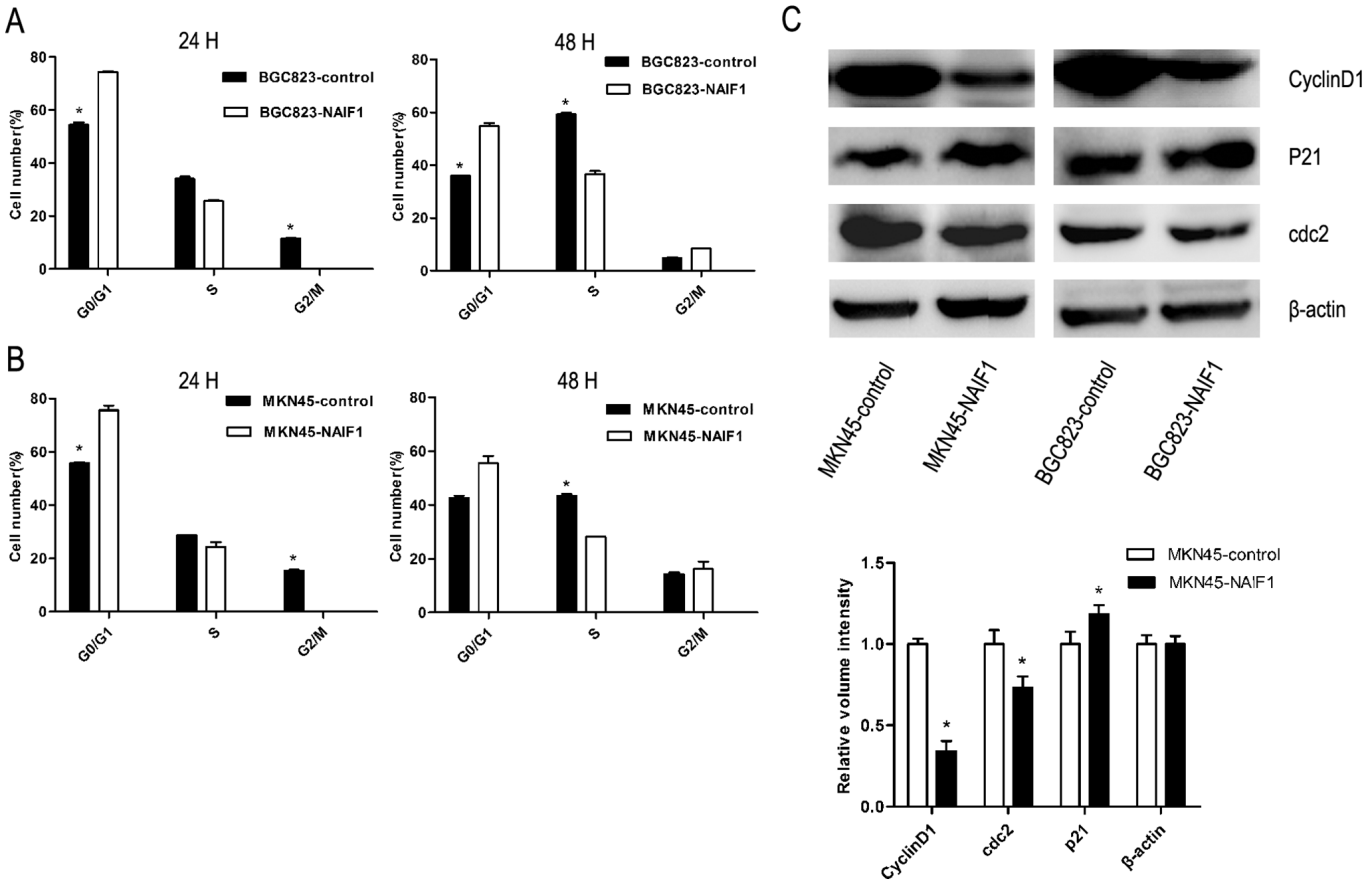
NAIF1 induced cell cycle arrest at G1/S phase. (A) and (B) columns show the different cell cycle distribution between cells transfected with NAIF1 and with the GFP vector 24 h or 48 h after transfection in (A) BGC823 cells and (B) MKN45 cells. To ensure the right population, GFP-positive population was analyzed. *Significant difference (*P*<0.05). (C) Western blotting shows the change in cell cycle regulating proteins associated with NAIF1 induced G1/S phase cell cycle arrest. β-actin was used as the control. Quantified data are shown as the mean ± SD (n = 3); Significant difference from the NAIF1 overexpressing group, Student's t test, *P<0.05.

The cell cycle profile of gastric cancer cells under the effect of NAIF1 was investigated next. GFP-positive cells were analyzed to ensure the correct population was used (Figure S2). Flow cytometric analysis demonstrated that NAIF1 induced a significant change in the distribution of the cell cycle: an increase of the cell population in the G0/G1 phase and a decrease in the S phase was observed in cells overexpressing NAIF1 compared with cells transfected with the empty vector, both after transfection 24 h and 48 h. As shown in Figure. 2A, flow cytometry revealed that 24 h post transfection, approximately 54% of BGC823 cells transfected with pEGFP-N1 vector were in the G0/G1 phase, and 34% and 12% of cells were in S phase and G2/M phase, respectively. On the other hand, in BGC823 cells transfected with the pEGFP-N1-NAIF1 construct there was a definite increase in the percentage of cells in G0/G1 phase (74%), as well as a decrease in the percentage of cells in S phase (26%) and G2/M phase (0%). Forty-eight hours post transfection, approximately 36% of the BGC823 control cells were in G0/G1 phase, and 59% and 5% of cells are in S phase and G2/M phase respectively. In BGC823 cells transfected with the pEGFP-N1-NAIF1 construct, approximately 55% of cells were in G0/G1 phase, and 37% and 8% of cells were in S phase and G2/M phase, respectively. In MKN45 cells, 24 h following transfection, approximately 56% of cells transfected with the pEGFP-N1 vector were in G0/G1 phase, and 29% and 15% of cells were in S phase and G2/M phase, respectively. Approximately 76% of MKN45 cells transfected with the pEGFP-N1-NAIF1 construct were in G0/G1 phase, and the percentages of cells in S phase and G2/M phase were decreased to 24% and 0%, respectively. Forty-eight hours after transfection, approximately 43% of the MKN45 control cells were in G0/G1 phase, and 43% and 14% of cells were in S phase and G2/M phase, respectively. In MKN45 cells transfected with the pEGFP-N1-NAIF1 construct, approximately 56% of cells were in G0/G1 phase, and 28% and 16% of cells were in S phase and G2/M phase, respectively (Figure. 2B). These results clearly demonstrate that overexpression of NAIF1 induced cell cycle arrest at G1/S phase of gastric cancer cells BGC823 and MKN45.

To clarify the mechanism by which NAIF1 induced cell cycle arrest, we examined the possible roles of cyclinD1, p21 and cdc2 proteins, which are important in cell cycle regulation in G1/S phase. Forty-eight hours after transfection, the expression levels of cyclinD1 and cdc2 were decreased compared with the control cells, both in BGC823 and MKN45 cells. Additionally, the expression levels of p21 were increased (Figure. 2C).

### Comparative proteomic analysis of the gastric cancer cell line MKN45 with or without extra expression of NAIF1 protein

**Figure 3:**
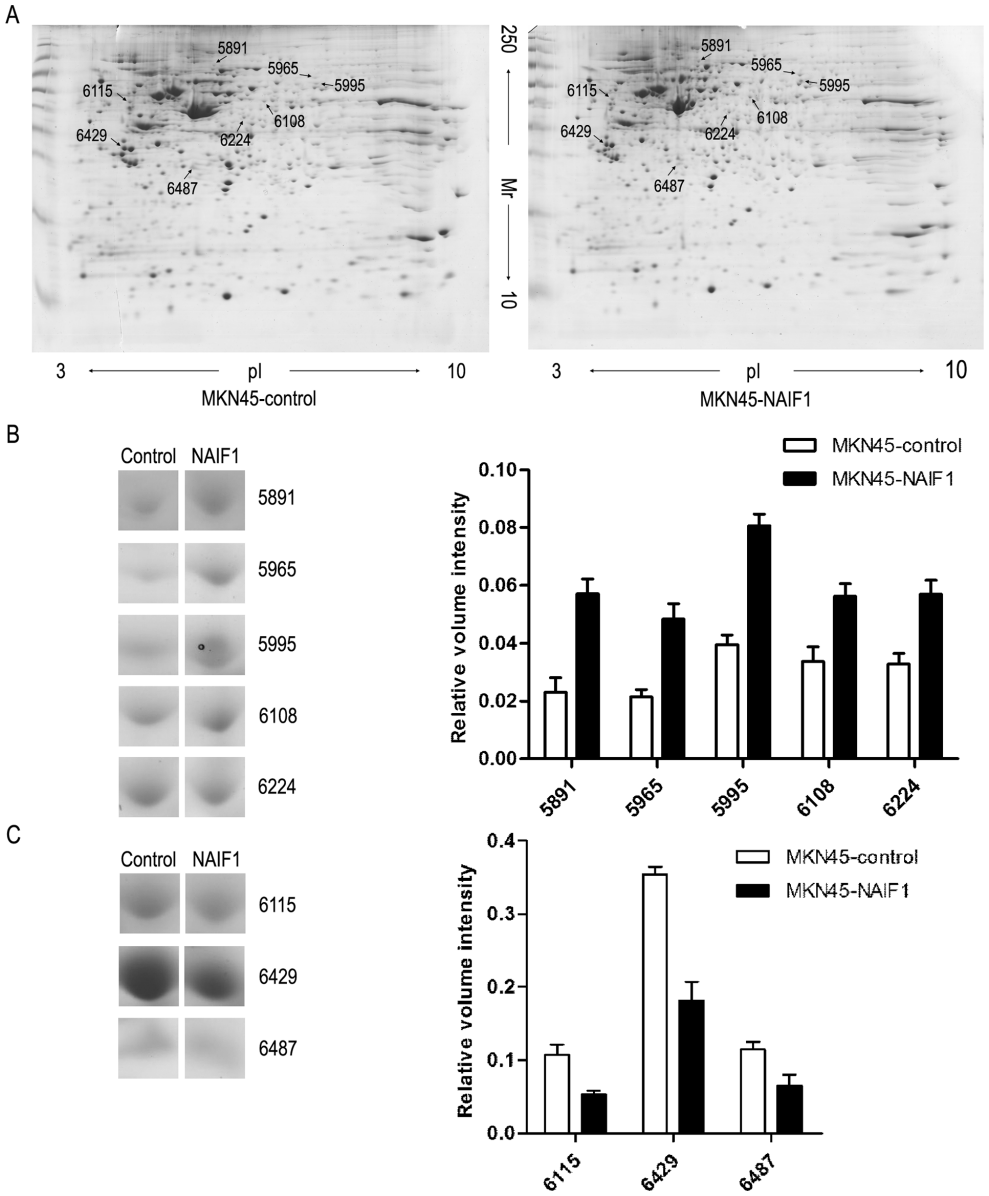
Proteomic analysis of MKN45 cells overexpressing NAIF1. (A) Representative 2-DE maps of MKN45 cells transfected with control plasmid or NAIF1. (B) and (C) Megascopic pictures and relative volume intensity of differential expressed proteins. (B) Represents the up-regulated protein points while (C) represents the down-regulated proteins.

The protein profiles of MKN45 cells transfected with the pEGFP-N1-NAIF1 constructs as the treated group or with the pEGFP-N1 vector as the control group were measured by 2-DE 48 h after transfection. In total, over 700 spots were resolved, of which 11 proteins was >1.5-fold differentially expressed as determined by analysis with Imagemaster version 5.0. The differentially expressed protein spots were excised from the gel, and 8 proteins were identified by MALDI-TOF/TOF analysis. Representative treated and control group 2-DE gel maps are shown in Figure. 3A and the differentially expressed protein spots are marked. Megascopic 2-DE gel pictures of each of the differentially expressed protein spots are represented in Figure. 3B and 3C. Five of the proteins were up-regulated in the treated group including NADH dehydrogenase (ubiquinone) Fe-S protein 1 (NADUSF1), chaperonin containing TCP1 subunit 3 (TCP1-γ), thioredoxin reductase 1 (TXNRD1), proteasome 26S subunit 2 (PSMC2) and proteasome 26S subunit 13 (PSMD13). 3 proteins were down-regulated, including ribonuclease inhibitor 1 (RNH1), 14-3-3 protein epsilon isoform (14-3-3 ε) and apolipoprotein A-I binding protein (APOAIBP). Table 2 depicts specific information on these proteins and their level of differential expression.

**Table 2:** Information on indentified proteins.

Spot No.	Protein AC	Protein description	MASCO T score	Theortical Mr(kDa)/pI	Change fold
5891	gi|316983156	NADH-ubiquinone oxidoreductase 75 kDa subunit	162	74.393/5.61	+2.48
5965	gi|14124984	Chaperonin containing TCP1, subunit 3	67	60.934/6.10	+2.25
5995	gi|49168498	TXNRD1	99	55.281/6.07	+2.04
6108	gi|4506209	26S protease regulatory subunit 7 isoform 1	212	49.002/5.71	+1.67
6115	gi|15029922	RNH1 protein	162	50.104/4.83	−2.02
6224	gi|157502193	26S proteasome non-ATPase regulatory subunit 13 isoform 1	87	43.203/5.53	+1.74
6429	gi|374074366	14-3-3 protein epsilon	107	29.326/4.63	−1.95
6487	gi|21068652	apolipoprotein A-I binding protein	128	32.010/7.56	−1.75

AC: accession number.

The “+” and “−” indicated the up-regulated and down-regulated proteins in MKN45 cells overexpressing NAIF1 compared with control, respectively.

### Validation of differentially expressed proteins

**Figure 4:**
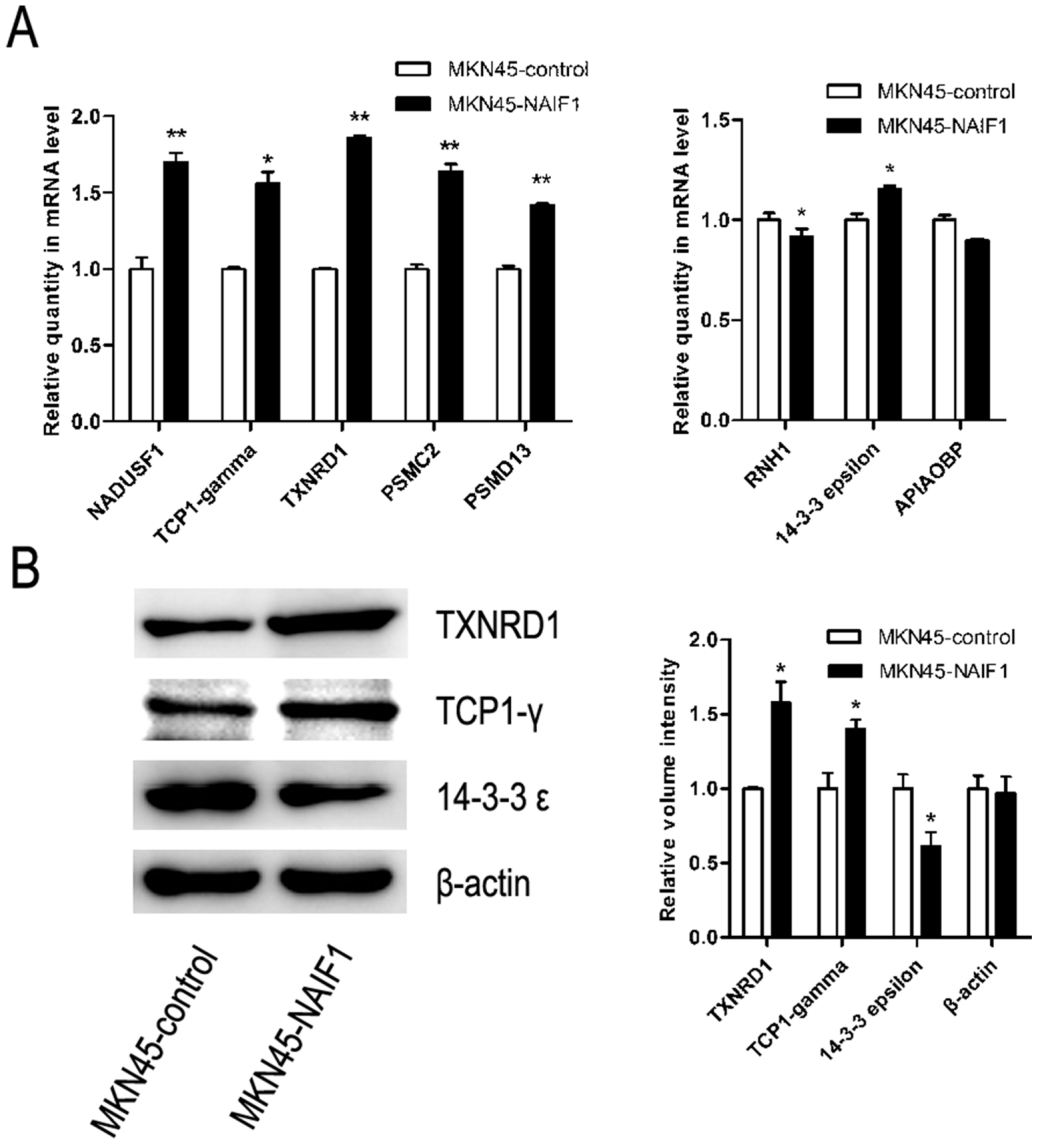
Verification of the 2-DE results. (A) Verification of the 2-DE results by real-time qPCR. *Significant difference (*P*<0.05) **Significant difference (*P*<0.001). (B) Verification of the 2-DE results by western blotting and the relative volume intensity of differential expressed proteins, using β-actin as control. *Significant difference (*P*<0.05).

Real-time qPCR and western blotting were performed to verify the 2-DE results. Gene expression levels of the identified proteins were analyzed by real-time qPCR and are consistent with the 2-DE results, except for one protein, 14-3-3ε (Figure. 4A). Three proteins were selected based on their importance and likely functions for further analysis by western blotting to verify the 2-DE results. The results show that the protein expression levels of TXNRD1 and TCP1-γ were significantly increased in the NAIF1 group compared to the control group (*P*<0.05), while the protein expression level of 14-3-3ε was significantly decreased in the NAIF1 group (*P<*0.05) (Figure. 4B). Overall, the western blot results are consistent with the 2-DE results.

## Discussion

Several studies have been performed to describe the NAIF1 gene; however, little is known about the proteomic profile NAIF1 is overexpressed. The aim of the present study was to detect and identify proteins whose expression is changed in MKN45 cells overexpressing NAIF1. Further, evidence is provided to explain how NAIF1 functions in inducing cell apoptosis and other activities. A 2-DE strategy was employed in this study because of its convenient methodology and high efficiency. In total, we detected and identified 5 proteins that were up-regulated and 3 proteins that were down-regulated in MKN45 cells overexpressing NAIF1 compared to the control group. The identified proteins involve a number of physiological activities, such as protein degradation, control of cell redox homeostasis, cell cycle progression, cytoskeleton regulation, cellular stress response, apoptosis and regulation of metabolism. The cell cycle profile of gastric cancer cell lines, BGC823 and MKN45 was also investigated, and our results demonstrated that overexpression of NAIF1 may induce cell cycle arrest at G1/S phase via altering the expression levels of cell cycle regulation proteins cyclinD1, cdc2 and p21.

Results from western blotting and reverse transcriptional PCR demonstrated that NAIF1 was minimally expressed in gastric cancer cells. The data presented herein is analogous to Luo's finding in tissues [10]. Moreover, we confirmed that NAIF1 is a nuclear protein in the gastric cancer cell lines MKN45 and BGC823. Since genomic DNA mainly exists in the nucleus, this data suggests that NAIF1 may function in the nucleus by interacting with genomic DNA and nucleoprotein in order to affect the expression of genes and proteins comprehensively. For this reason we performed proteomic analysis to investigate the proteomic profiling of MKN45 cells overexpressing NAIF1.

Abnormal regulation of cell cycle is a remarkable characteristic of cancer cells [11]. Studies have demonstrated that many small molecules can activate cell cycle checkpoints and induce cell cycle arrest, which allow cells to repair defects. However, unsuccessful repairation may lead to apoptosis [12]–[15]. Our results show that NAIF1 induces cell cycle arrest at G1/S phase. Transition from G1 to S phase requires the activation of assembly of cyclinD-Cdk4/6 and cyclinE-cdc2 (also known as Cdk1). Meanwhile, p21 inhibits the activity of cdc2 and mediates assembly and activation of cyclinD-cdk4/6 in the cytoplasm, as well as inhibiting their activity in the nucleus [16]. In this study, western blotting analysis detected that protein expression levels of cyclinD1 and cdc2 were decreased, while the protein expression level of p21 was increased in cells overexpressing NAIF1. These results suggest that G1/S cell cycle arrest induced by NAIF1 is mediated through the p21 and cyclinD1 pathway. In addition, we demonstrated that 48 h after transfection, the population of apoptotic cells increased approximately 10% in cells overexpressing NAIF1 as a result of cell cycle arrest (Figure S3).

26S proteasome is a conservative organelle in all eukaryotic cells and it is responsible for intracellular protein degradation [17]. Proteins that are degraded by 26S proteasome are involved in a series of biological processes including apoptosis, cell cycle, and growth and regulating the expression of many genes which in turn regulate other processes [18]. Disturbance of the protein degradation equilibrium leads to the origination and development of cancer. Thus, 26S proteasome is an attractive cancer therapy target. Here, we found that two subunits of the 26S proteasome, PSMC2 and PSMD13, were both up-regulated in MKN45 cells overexpressing NAIF1. PSMC2 and PSMD13 are subunits of the 19S proteasome, which is the regulatory particle (RP) of the 26S proteasome. PSMC2 is an ATPase while PSMD13 is a non-ATPase. Some researchers believe that 26S proteasome inhibition may lead to apoptosis of carcinoma cells, and proteasome inhibitors could be used to treat cancer [19]–[21]. However, a study by Tan *et al.* suggests that tumor necrosis factor (TNF)-α activates the 26S proteasome system by up-regulating the expression levels of the 26S proteasome subunits [22]. TNF-α is a well known cytokine which can induce apoptosis in a range of cancer cells, and now it is used in the clinic as a regional treatment of locally advanced soft tissue sarcomas and metastasis melanomas to avoid of amputation limbs [23]. Like TNF-α, NAIF1 also has the ability to induce apoptosis, which implies that the 26S proteasome may be involved in the apoptosis process induced by NAIF1.

Our data also demonstrate that two proteins, TXNRD1 and NDUFS1, are up-regulated by NAIF1. TXNRD1 regulates the redox state of protein thiols in mammalian cells, and functions in both promoting and preventing cancer in different kinds of carcinomas [24]–[27]. There have been no studies to investigate the role of TXNRD1 in gastric cancer. In our opinion, TXNRD1 may participate in the suppression of gastric cancer genesis or the up-regulation of TXNRD1 may be an adaptive mechanism in response to oxidative stress generated by overexpression of NAIF1. The NDUFS1 gene encodes a 75 kDa Fe-S subunit, which is one of the seven mitochondrial subunits of complex I [28]. Complex I is the largest of the respiratory chain enzymes and deficiency of complex I is the major cause of a series of inborn mitochondrial diseases, such as Leigh syndrome [29]. In addition, the 75 kDa subunit of complex I is a caspase substrate, which is involved in the mitochondrial apoptosis pathway. Caspase cleavage of NDUFS1 is required for several mitochondrial changes associated with apoptosis, including ATP levels, ROS production, and loss of plasma membrane integrity and so on [30]. Since NAIF1 induces apoptosis through the mitochondrial pathway [8], we hypothesize that the up-regulation of NDUFS1 participates in the apoptosis process induced by NAIF1.

TCP1-γ has been identified as a chaperonin in eukaryotic cytosol. Previous research has found that TCP1-γ is significant expressed in tumors of hepatocellular carcinoma compared to the paired adjacent non-malignant tumor tissues [31], [32]. However, the relationship between TCP1-γ and gastric cancer is still unknown and further study is needed.

On the other hand, we identified three proteins, 14-3-3ε, RNH1 and APOAIBP, that were down-regulated in MKN45 cells overexpressing NAIF1. 14-3-3 is a protein family consisting of multifunctional proteins that bind to and modulate the functions of a wide range of cellular proteins. Further, they participate in diverse biological processes, including cell cycle checkpoint regulation, apoptosis, and cell growth control [33]. Several studies have reported that 14-3-3ε expression is increased in lung cancer, hepatocelluar cancer, breast cancer and vulvar squamous cell carcinoma [34]–[37]. The only exception to this increased 14-3-3ε expression is in gastric cancer tissues, where the expression is decreased [38]. In addition, previous studies have demonstrated that up-regulation of 14-3-3ε may prevent Bad-triggered apoptosis in endothelial cells [39], and non-steroidal anti-inflammatory drugs can induce colorectal cancer cell apoptosis by suppressing 14-3-3ε expression [40]. Moreover, a histopathological study reported that in 114 hepatocellular carcinoma patients, elevated expression of 14-3-3ε was significantly associated with a high risk of metastasis and decreased survival rates [35]. Cellular experiments confirmed that increased 14-3-3ε expression induces hepatocellular carcinoma cell migration and promotes epithelial-mesenchymal transition (EMT) by inducing Zeb-1 and Snail expression [41]. To conclude, 14-3-3ε is a remarkable oncogene that participates in the development of several tumors and plays an important role in apoptosis and metastasis. Here, we found that 14-3-3ε is down-regulated in MKN45 cells overexpressing NAIF1, suggesting that 14-3-3ε may be involved in the apoptosis process induced by NAIF1. Moreover, our data implies that besides inducing apoptosis and cell cycle arrest, NAIF1 may play a role in cell migration and metastasis. Our results demonstrate that the protein expression levels of 14-3-3ε were decreased, while the RNA levels were increased. These results reveal that NAIF1 regulates the expression levels of 14-3-3ε posttranslationally. Our data showing the differential expression levels of 14-3-3ε are contrary to Leal's [38], which may be due to the difference between cells and tissues, or may be attributed to the bias in specimen selection.

RNH1 is a cytosolic protein that inhibits RNases and forms high-affinity heterodimers with them to play a controversial role in angiogenesis [42]. There has been an increase in research studies to investigate the relationship between RNH1 and cancer. Kim *et al.* demonstrated that RNH1 binds to the Drosha complex directly to promote miRNA21 processing, which is a cancer initiation process [43]. Another study using genetic knockdown and overexpression assays revealed that RNH1 is necessary and sufficient to induce resistance to histone deacetylase inhibitors (HDACis) in gastric cell lines [44], suggesting that inhibition of RNH1 may coordinate measures in clinical chemotherapy. Our research found NAIF1 inhibits the expression of RNH1, which suggests that NAIF1 may collaborate with chemotherapy drugs in the clinical treatment of gastric cancer.

The apoA-I binding protein was first identified by screening a human liver two-hybrid cDNA library by using mature apoA-I protein. These results demonstrate that the apoA-I binding protein interacts with the apoA-I protein and thus plays a role in the regulation of the stability, lipid transport and metabolism of high-density lipoproteins (HDL) [45]. No studies have been performed to elucidate the role of APOAIBP in human cancer, so we could not hypothesize its precise role in gastric cancer.

In conclusion, we found NAIF1 was minimally expressed in gastric cancer cell lines, and confirmed that NAIF1 is a nuclear protein. Moreover, we demonstrated that NAIF1 induces cell cycle arrest at G1/S phase by regulating the protein expression of cyclinD1, cdc2 and p21. We screened and identified proteins whose expression was altered due to overexpression of NAIF1 in the gastric cancer cell line MKN45 using comparative proteomic technology, and the results were further verified through real-time qPCR and western blotting. The elevated expression of PSMC2, PSMD13, TXNRD1 and NDUSF1, and the decreased expression of 14-3-3ε and RNH1 may participate in apoptosis induced by NAIF1. Taken together, our results provide useful information to explain the mechanism of how NAIF1 induces apoptosis. Therefore, NAIF1 may have therapeutic potential in the diagnosis and treatment of gastric cancer.

## Supporting Information

Figure S1 **Subcellular distribution of NAIF1 in BGC823 cells.** BGC823 cells were transfected with the NAIF1-GFP fusion construct or GFP vector for 48 h and cells were harvest and stained with DAPI. Photographs were obtained using a Leica Microsystems Heidelberg GmbH microscope. The NAIF1-GFP fusion protein was localized only in the nuclei while GFP was distributed in the entire cell.
(TIF)

Figure S2 **Gates were set to ensure that right population was measured.** For the flow cytometry analysis, we set three gates to ensure we measured the correct cell populations. Gate R1 excluded cell debris, Gate R2 scaled the GFP positive cells, which were cells that had been transfected successfully, and Gate R3 excluded the aggregates.
(TIF)

Figure S3 **NAIF1 induced cell apoptosis significantly 48 h after transfection.** Gastric cancer cell lines BGC823 and MKN45 were transfected with the NAIF1-GFP construct or the GFP vector for 24 or 48 h. The apoptosis ratio of GFP positive cells was measured. Q2 combined with Q4 represents the percentage of apoptotic cells among total cells. Forty-eight hours after transfection, the apoptosis ratio of BGC823 cells overexpressing NAIF1 was 31.4% while that of BGC823 control cells was 22.9%; for MKN45 cells, the apoptosis ratio was 30.9% for cells overexpressing NAIF1 and 21.2% for control cells.
(TIF)
