# Supplementary figures and images for: Overexpression of Nuclear Apoptosis-Inducing Factor 1 Altered the Proteomic Profile of Human Gastric Cancer Cell MKN45 and Induced Cell Cycle Arrest at G1/S Phase
Source: PLoS One. 2014 Jun 13;9(6):e100216. doi: 10.1371/journal.pone.0100216 (PMC4057436; doi:10.1371/journal.pone.0100216)

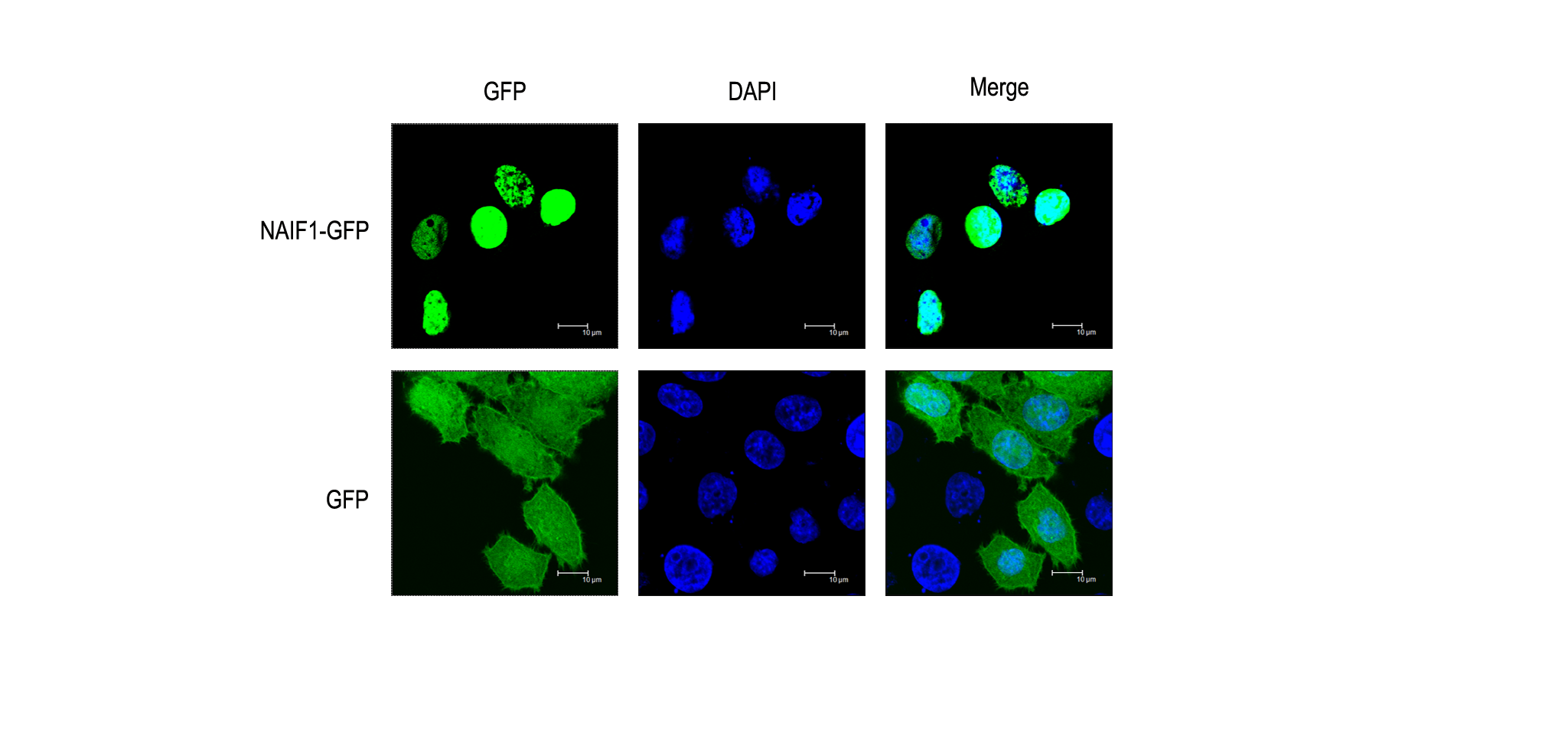

Supplement: Figure S1 — Subcellular distribution of NAIF1 in BGC823 cells. BGC823 cells were transfected with the NAIF1-GFP fusion construct or GFP vector for 48 h and cells were harvest and stained with DAPI. Photographs were obtained using a Leica Microsystems Heidelberg GmbH microscope. The NAIF1-GFP fusion protein was localized only in the nuclei while GFP was distributed in the entire cell. (TIF) [file pone.0100216.s001.tif]

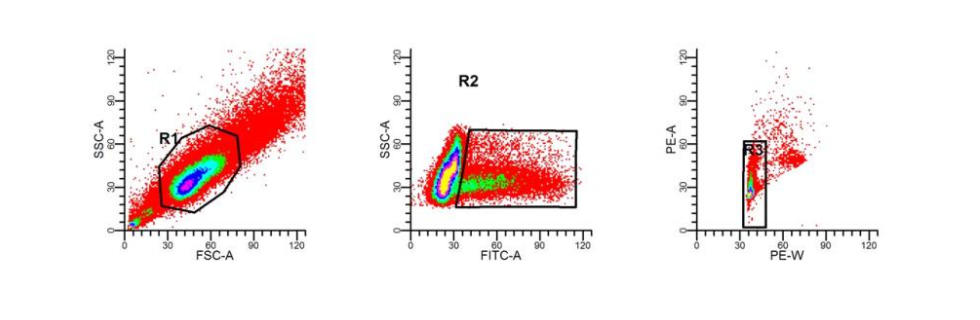

Supplement: Figure S2 — Gates were set to ensure that right population was measured. For the flow cytometry analysis, we set three gates to ensure we measured the correct cell populations. Gate R1 excluded cell debris, Gate R2 scaled the GFP positive cells, which were cells that had been transfected successfully, and Gate R3 excluded the aggregates. (TIF) [file pone.0100216.s002.tif]

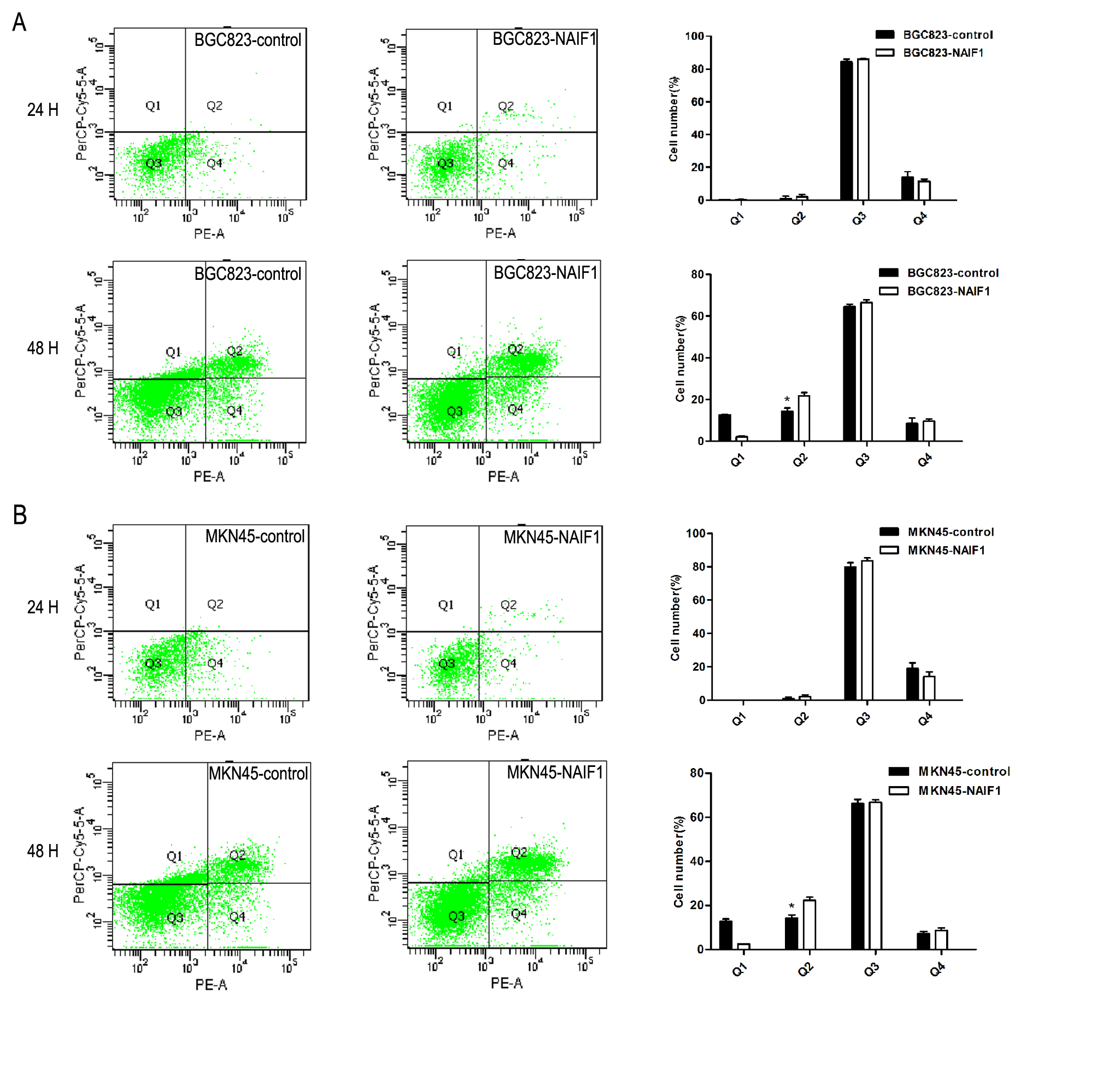

Supplement: Figure S3 — NAIF1 induced cell apoptosis significantly 48 h after transfection. Gastric cancer cell lines BGC823 and MKN45 were transfected with the NAIF1-GFP construct or the GFP vector for 24 or 48 h. The apoptosis ratio of GFP positive cells was measured. Q2 combined with Q4 represents the percentage of apoptotic cells among total cells. Forty-eight hours after transfection, the apoptosis ratio of BGC823 cells overexpressing NAIF1 was 31.4% while that of BGC823 control cells was 22.9%; for MKN45 cells, the apoptosis ratio was 30.9% for cells overexpressing NAIF1 and 21.2% for control cells. (TIF) [file pone.0100216.s003.tif]
